# EP36 Caecal vasculitis: a rare and dangerous complication of Sjögren’s syndrome

**DOI:** 10.1093/rap/rkaa052.035

**Published:** 2020-11-03

**Authors:** Jessica Ellis, Keziah Austin, Harsha Gunawardena

**Affiliations:** North Bristol NHS Foundation Trust, Bristol, United Kingdom

## Abstract

**Case report - Introduction:**

We present the case of a patient with Primary Sjögren’s syndrome (pSS) who presented via the general surgical take with an acute abdomen, necessitating emergency subtotal colectomy. Histology demonstrated vasculitis of the caecum, and in combination with elevated type II cryoglobulins and unmeasurable complements, a diagnosis of cryoglobulinaemic vasculitis was made. Vasculitis has a recognised association with pSS, particularly in the context of elevated cryoglobulins, but bowel involvement is rare. Clinicians involved in the care of pSS patients should be alert to the possibility of rare but severe multi-system manifestations, due to their high burden of morbidity and mortality.

**Case report - Case description:**

This 54-year-old female is under the care of Rheumatology for pSS. She initially presented with sicca symptoms, fatigue, arthralgia, and parotid swelling for which she had undergone a superficial parotidectomy. She had longstanding constitutional symptoms of night sweats, weight loss and fever. She also reported chronic constipation as well as a photosensitive urticarial rash. At diagnosis, her ENA panel demonstrated SS-A, SS-B and SCLER-70 positivity with type II cryoglobulins of 0.56 and hypocomplementemia (C4 < 0.01). At this stage there were no clinical or laboratory markers of end organ damage. Initial treatments included Hydroxychloroquine and Azathioprine with recent switch to Methotrexate due to inefficacy.

2 years later, she presented emergently via the general surgical team with a one-day history of generalised abdominal pain and vomiting. On examination she had right lower quadrant peritonism. CT scan demonstrated severe caecal colitis with associated ascites, requiring emergency sub-total colectomy. Histology from the resected bowel demonstrated ischaemia with numerous foci of submucosal vasculitis.

On inpatient Rheumatology review there were no cutaneous, pulmonary, musculoskeletal features of vasculitis. She had reduced pinprick sensation to her feet, associated with allodynia. Laboratory tests showed a haemoglobin of 106, platelets of 866 and albumin of 27 (all markers felt to reflect recent critical illness). Her eGFR was 71 (from a baseline of 90) with urine PCR of 11.7 but no blood. Faecal calprotectin was normal. EBV, CMV, Hepatitis B and C and HIV were negative. Repeat immunology confirmed a type II cryoglobulinemia of 0.95 and C4 of 0.01. Following MDT discussion with colleagues in both Gastroenterology and Renal medicine it was agreed that her colitis likely represented a cryoglobulinaemic vasculitis secondary to pSS.

She was treated with oral prednisolone and six intravenous pulses of cyclophosphamide. After six months she is symptomatically improved with negative cryoglobulins and normal complement.

**Case report - Discussion:**

pSS is an immune-mediated condition classically associated with sicca symptoms commonly affecting the eyes and mouth. These symptoms derive from immune mediated inflammation and damage of secretory glands and resultant drying of mucosal surfaces.

However, extra-glandular involvement in pSS is common, both at presentation and later in the disease course. Organ systems most associated include joints, lungs, skin, and peripheral nerves. However, involvement of other organ systems, particularly gastrointestinal or pulmonary are associated with significant morbidity and mortality.

Gastrointestinal involvement in pSS is well recognised and encompasses manifestations from dysphagia to pancreatitis. Symptoms related to irritable bowel syndrome, including constipation as in our patient, are common but generally follow a benign course. Our patient never experienced any symptoms suggesting inflammation of the bowel, such as diarrhoea or rectal bleeding prior to her acute presentation.

Several prognostic markers have been proposed for pSS, including SS-A/ SS-B positivity, hypocomplementemia and cryoglobulinemia. These immunological markers, particularly low C4, are implicated in an increased risk of developing vasculitis. These markers were present in our patient at the time of diagnosis; at this point there were no clinical features suggestive of vasculitis.

Vasculitis in pSS, when seen, is most associated with the skin and kidneys, although involvement of the small bowel has been observed. Ileal biopsies for our patient, performed prior to immunosuppression, were normal suggesting that in this case the vasculitis was limited to the large bowel. Cryoglobulinaemic vasculitis, secondary to mixed cryoglobulinemia, is seen in association with connective tissue diseases, most commonly pSS. Gastrointestinal involvement has also been recognised in this context, but again is uncommon, compared to other vasculitides.

After immunosuppression, our patient’s cryoglobulins resolved and she has remained clinically stable. Her case provides an important lesson regarding the possibility of severe extra-glandular vasculitic manifestations in pSS patients.

**Case report - Key learning points**
 

- Systemic involvement in pSS is relatively common
- Immunological markers exist which can prognosticate both the risk of systemic involvement and the development of vasculitis
- Caecal vasculitis is rarely seen in pSS; when present it carries a large burden of morbidity and mortality
- Increasing awareness of pSS and its systemic manifestations is essential to facilitate better recognition of unusual presentations.

